# A Prospective Study of the Outcome of Patients with Limb Trauma following the Haitian Earthquake in 2010 at One- and Two- Year (The SuTra2 Study) 

**DOI:** 10.1371/currents.dis.931c4ba8e64a95907f16173603abb52f

**Published:** 2013-07-05

**Authors:** Marie Christine Delauche, Nikki Blackwell, Hervé Le Perff, Nezha Khallaf, Joël Müller, Stéphane Callens, Thierry Allafort Duverger

**Affiliations:** The Alliance for International Medical Action (ALIMA); The Alliance for International Medical Action (ALIMA); The Alliance for International Medical Action ALIMA, Fann Résidence, BP15530The Alliance for International Medical Action (ALIMA); Université Lille Nord de France, LEM UMR 8179 CNRS; Université Lille Nord de France - Université d'Artois; University of ArtoisLEM UMR 8179 CNRS; The Alliance for International Medical Action (ALIMA)

## Abstract

Background Severe limb trauma is common in earthquake survivors. Overall medium term outcomes and patient-perceived outcomes are poorly documented. Methods and Findings The prospective study SuTra2 assessed the functional and socio-economic status of a cohort of patients undergoing surgery for limb injury resulting in amputation (A) or limb preservation (LP) one year and two years after the 2010 Haiti earthquake. 305 patients [A: n=199 (65%), LP: n=106 (35%)] were evaluated. Their characteristics were: 57% female; mean age 31 years; 74% of principal injuries involved the lower limb; 46% of patients had an additional severe injury; 60% had fractures, of which two-thirds were compound or associated with severe soft tissue damage; 15% of amputations were traumatic. At 2 years, 51% of patients were satisfied with the functional outcome (A: 52%, LP: 49%, ns). Comparison with the 1-year status indicates a worsening of the perceived functional status, significantly more pronounced in amputees, and an increase in pain complaints, mainly in amputees (62% and 80% of pain in overall population at 1- and 2-year respectively). Twenty eight percent (28%) of LP and 66% of A considered themselves as “cured”. 100% of LP and 79% of A would have chosen a conservative approach if an amputation was medically avoidable. Two years after the earthquake, 23·5 % of patients were still living in a tent, 30% were working, and 25·5% needed ongoing surgical management. Conclusions Only half the patients with severe limb injuries, whether managed with amputation or limb preservation, deemed their functional status satisfactory at 2 years. The patients’ perspective, clearly favors limb conservative management whenever possible. Prolonged care and rehabilitation are needed to optimize the outcome for earthquake survivors with limb injuries. Humanitarian respondents to catastrophes have professional and ethical obligations to provide optimal immediate care and ensure scrupulous attention to long-term management. Keywords Haiti earthquake, limb injury, two-year outcome, patients’ perspective, amputation, limb salvage

## Introduction

Many wounded earthquake survivors have limb injuries; resource constraints may compromise their optimal care. The decision to amputate is always difficult while the feasibility of limb preservation in the emergency response phase is uncertain. Functional disabilities due to limb injuries may jeopardize the return to work of injured individuals, who are likely to struggle economically and become a burden on their families and communities^1^. Finally, lower limb (LL) reconstruction has been shown more acceptable psychologically to patients with severe trauma compared with amputation even though the physical outcome for both management pathways was similar^2^. After the 12^th^ January 2010 Haiti earthquake, about 1,200-1,500 amputations were performed for limb injuries^3^. Protracted rehabilitation of amputees as well as of patients undergoing limb reconstruction is unanimously considered crucial^4^^,^ 5 ^,^ 6 ^,^ 7.

Reports on victim management and outcome after mass catastrophe^8^^,^ 9 including those on the recent Haiti disaster 3 ^,^ 7 ^,^ 10 ^,^ 11 ^,^ 12 ^,^ 13 ^,^ 14 ^^,^^ 15 ^,^ 16 ^,^ 17 ^,^ 18 ^,^ 19 ^,^ 20 rarely extend more than six months after the tragedy. The non-governmental organization (NGO) Alliance for International Medical Action (ALIMA, France) in coordination with the Lille Economics Management (LEM, France) conducted a prospective observational cohort study 1 year and 2 years after the earthquake (SuTra^2^ Project). The aim was to document the medium-term outcome of individuals with severe limb injuries sustained during the 2010 earthquake in Haiti, treated with either limb amputation or limb surgical preservation with a special focus on the patient’s perspective. It was also planned to evaluate the impact of the surgical treatment on outcomes.

## Methods

**Patients and study design**

Patients with limb injuries due to the earthquake, living in Port au Prince or its suburbs and who underwent limb surgery resulting in either limb amputation (A) or limb preservation (LP), were recruited by phone. They were contacted from database listings issued by: 1) The Clinique Lambert (Pétion-Ville, Haiti); two NGOs: 2) Handicap International (HI) and 3) Bangladesh Rural Advancement Committee (BRAC), and 4) a local organization, l’Union des Jeunes Victimes du Séisme (UJVS) (Table 1). Limb surgery was defined as any surgical procedure on a limb that required general or regional anesthesia, whatever the delay from the initial injury. When a patient had injuries involving more than one limb, the principal injury according to the patient, was considered as the main injury. Associated severe injuries were named “additional” and could involve any part of the body.

**Procedures**

Patients fulfilling the above criteria, who agreed to participate in the study, were included in the 1-year assessment from January 21^st^ to March 29^th^ 2011, and in the 2-year assessment from January 23^rd^ to March 29^th ^2012. Recruitment was stopped when everyone on the database listings had been contacted. Medical, quality of life (SF 36)^21^^,^ 22 and socio-economic data were collected through pre-established case report forms (CRF) in French. Demographics, history of the injury, surgical treatment, duration of hospitalization and physiotherapy, infection, pain (any pain and pain intensity through a visual analogue scale - VAS -), clinical examination of the injured limb (s), functional assessment (according to a 4-point scale; not satisfied, poorly satisfied, satisfied, very satisfied) and need for additional care were recorded. The socio-economic questionnaire explored the circumstances of the trauma, level of education, housing, family status and the theoretical patient preference between amputation and limb preservation (question addressed in 2011). To decrease the variability of the medical assessments, the number of examiners was restricted to three: a physician who examined amputees and nearly all the patients with limb reconstruction and two physiotherapists (one in 2011, one in 2012) trained in the study method by the physician, with a Creole translator when necessary. The physician reviewed all the patients’ charts and the medical CRFs. Three Haitian psychologists (2 in 2011, and 2 in 2012) administered the validated French SF36 and socio-economic questionnaires in patients over 15 years. When necessary, patients were referred to a specialised centre for surgical, physiotherapy or prosthesis advice, or for a psychological consultation. Patients or child's parents (caretaker) provided written informed consent and received compensation for travel expenses. The study received Ethics Committee approval from the Haitian Ministry of Health in both 2011 and 2012.The protocol is available through the link http://www.alimaong.org/wp-content/uploads/2012/12/SuTra-protocol-research-EN-1.pdf. The study is registered at ClinicalTrials.gov (registration number: NCT01779011).

**Data handling and statistical analysis**

In many cases, especially in amputees, the history reflected the patients’ description because no substantial patient record was available. Whenever possible, any information gathered from a patient chart, which was available for all the patients recruited via the Clinique Lambert (most LPs), was checked with the patient’s history. Radiographs at the time of the first surgical procedure were usually missing. Limb injuries were classified simply, indicating the presence of a fracture, closed or compound and/or presence of severe soft tissue damage with skin barrier impairment (SSTD). No severity scoring system could be applied retrospectively to the initial injuries. The main outcome criterion was an analysis of patients’ satisfaction with their functional status. Other outcome criteria were: satisfaction with the overall care, residual pain, need for additional care, resumption of previous physical activities, patient preference regarding their procedure, and employment status.

Descriptive analysis of quantitative and qualitative variables was performed for the overall population and according to the status A or LP, at 1-year and/or at 2-year, depending on the variable. As 76% of Haitians between 15- and 29-yr are single^23^, marital status was analyzed in the population over 29-yr. The main baseline characteristics (age, sex, proportion of A and LP, type and proportion of upper and lower limb injuries) of patients attending the 1- and 2-year visits were compared. Additional data analysis consisted of comparison of means (t-test, ANOVAs), and correlations. A subgroup analysis was conducted in 46 patients with lower limb injuries (A n= 23; LP: n=23), matched by age, sex, number of additional lesions and type of initial injury. SF36 scores were analysed according to Ware and Sherbourne^21^. Psychological (mean of the 4 psychological domains) and physical (mean of the 4 physical domains) subscales were also calculated (legend of Figure 2). Reliability, convergent and discriminating validities were measured and checked before applying the SF36 domains in the model. The SF 36 scores go from 0% to 100% (optimal)

## Results

***Baseline characteristics of the population***

Patient sources are given in Figure 1 and Table 1. Overall 305 patients were included in the study, 282 in the functional and socio-economic analysis at 1-year, 235 at 2-year; 212 patients attended both 1 and 2-year visits and 70 patients (24%) were lost to follow-up between 2011 and 2012. The majority of patients with LP (96%) were enrolled from the Clinique Lambert database. Overall, patients had procedures in 65 different surgical centres. The baseline characteristics of patients attending the 1-year visit and the 2-year visit were similar.

**Diagram of the patients’ selection:**
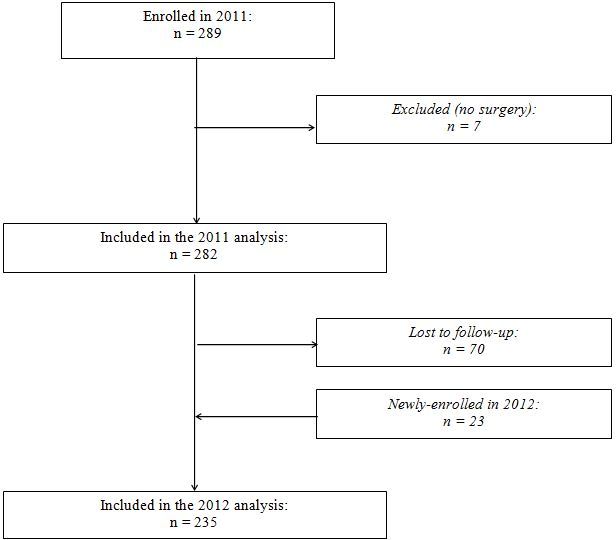

**Table 1. Studied populations, patients’ visits and source:** *Abbreviations:* LP: Patients with limb preservation; yr: year

	Amputees	L P	Total
All included (1 and / or 2-yr), n	199	106	305
Visit at 1-yr	188	94	282
Visit at 2-yr	152	83	235
			
Functional outcome population, n (%)			
1 and 2-yr	141 (71)	71 (67)	212 (70)
Lost of follow-up at 2-yr	47 (24)	23 (22)	70 (23)
Included at 2-yr	11 (5)	12 (11)	23 (7)
			
Patients source: n (%)			
Clinique Lambert	15 (8)	102 (96)	117 (39)
Handicap International	135 (68)	-	135 (44)
UJVS	35 (18)	-	35 (11)
Word of mouth	9 (4)	1 (1)	10 (3)
BRAC	5 (2)	3 (3)	8 (3)
			

The main characteristics of the overall population, A and LP subgroups are given in Table 2. Amputees and patients with LP differed according to age, mode of extrication, location and type of principal limb injury, and number of injuries. In general, amputees were younger, and a higher proportion of amputees were below 15-yr. They also had predominantly lower limb (LL) injuries, and more severe injuries as evidenced by the greater frequency of compound fractures or severe associated soft-tissue damages (and traumatic amputation). Patients with LP had more closed fractures and more additional injuries.

**Table 2. Baseline characteristics of the population:** *Abbreviations: *LP: limb preservation; n: number; sd: standard deviation; yr: year; hr: hour; LLI: Lower Limb Injury, ULI: Upper Limb Injury, SSTD: Severe Soft Tissue Damage; † p<0·05, ‡ p<0·01 comparison A vs LP. ^∆ ^:Age in 2011; ^a^: Population 1 year aged > 29 yr: overall n = 128, A: n = 78, LP: n = 50; ^b^: Population 1 year, patients > 15 yr: n=238, A n=153; LP n= 85; 2 missing values °: Patients with at least one additional injury located on another limb or any other part of the body

Characteristics		Amputees n = 199	LP n = 106	Overall n = 305
Female, n (%)		113 (57)	62 (58·5)	175 (57)
Age^∆^, yr, n (%)				
≤ 15		33 (17)	9 (8)	42 (14)
> 15 - ≤ 65		164 (82)	94 (89)	258 (84)
> 65		2 (1)	3 (3)	5 (2)
mean (sd)		29 (14)	35 (16)‡	31 (15)
Marital status^a^, n (%)				
Married or cohabitation		47 (60)	30 (60)	77 (60)
Education^b^, n (%)				
Primary study or illiterate		50 (33)	37 (44)	87 (36)
Entrapment^b^, n (%)				
Patients entrapped		102 (67)	46 (54)	148 (62)
Self-extrication,		10 (10)	11 (24) †	21 (14)
Duration of entrapment				
	- ≤ 6 hr	70 (68)	38 (82·5)	108 (73)
	- > 6 ≤ 24 hr	16 (16)	3 (6·5)	19 (13)
	- > 24 hr	16 (16)	5 (11)	21 (14)
Upper Limb Injury (ULI), n (%)		40 (20)	39 (37)	79 (26)
Forearm		11 (5)	18 (17)	29 (9)
Hand		15 (8)	12 (11)	27 (9)
Arm		12 (6)	2 (2)	14 (5)
Elbow		1 (0.5)	6 (6)	7 (2)
Shoulder		1 (0.5)	1 (1)	2 (1)
Lower Limb Injury (LLI), n (%)		159 (80)	67 (63)	226 (74)
Leg		80 (40)	21 (20)	101 (33)
Foot		62 (31)	14 (13)	76 (25)
Thigh		14 (7)	24 (22)	38 (12)
Knee		2 (1)	4 (4)	6 (2)
Hip		1 (0·5)	4 (4)	5 (2)
Multiple Injuries°				
N. patients, n (%)		86 (43)	55 (52)	141 (46)
Another limb, n (%)		35 (18)	25 (24)	60 (13)
N. additional injuries, mean (sd)		1·4 (0·7)	1·65 (0·8) †	1·5 (0·7)
Main Injury, n (%)				
Overall Fracture		104 (52)	81 (76) ‡	185 (60)
	- Closed	10 (5)	50 (47) ‡	60 (20)
	- Associated with SSTD	53 (26) ‡	10 (9)	63 (20)
	- Compound	41 (21)	21 (20) ‡	62 (20)
Crush Injury		40 (20) ‡	3 (3)	43 (14)
Traumatic Amputation		30 (15)	-	30 (10)
SSTD		20 (10)	7 (7)	27 (9)
Other		5 (2·5)	15 (14)	20 (7)

**Management.** The management of limb injuries (surgical procedures, hospital stays, physiotherapy) is given in Table 3. The delay to the first surgical procedure was shorter in amputees and only 3% of amputees had their first surgery performed beyond one month compared to 38% of patients with LP. Twenty nine percent of LP (29% ; 31/106) had limb injuries such as compound fractures or SSTD, which might have lead in this context to amputation. Conversely 15% of amputees (29 out of 199) had a previous attempt to preserve the limb. The rate of stump revision was 30% (61/199). Infections were commonest among amputees but chronic osteomyelitis was only observed as a complication of osteosynthesis. Hospital length of stay (cumulative) was significantly longer in amputees. Eighty nine percent (89%) of patients had access to physiotherapy, which lasted more than 3 months in 57% of them.

**Table 3. Therapeutic Management:** *Abbreviations: *LP: Limb preservation; n: number; sd: standard deviation; d: day; mo: month †: p<0·05, ‡: p<0·01 comparison A vs LP; •: Including traumatic amputation (n=30) ¶: Several surgical procedures possible under one anesthesia: all surgical procedures at first surgery: n=328 (A: n=205; LP: n=123) ¤: Delay to first surgery ≤ 30 days: n=253 (>30 days: A: n= 7, LP: n= 36) ^a^: Population assessed in 2011, 2 missing data; for the population assessed in 2012 (n=235): mean number of surgical procedures: A: n= 2·1 (1·5), LP: n = 3·5 (2·1) ‡, overall : 2·6 (1·8)

Management		Amputation n=199	LP n=106	Overall n=305
Delay to First Surgery¤, d, mean (sd)		6 (5·1)	11 (6·8) ‡	7 (5·9)
Type of First Surgery ¶, n				
Amputation		170	-	170
Osteosynthesis		2	46	48
External Fixator		7	30	37
Debridement		16	15	31
Other		10	32	42
Surgical Procedures^a^, n (%)				
≤ 2		147 (74) ‡	49 (47)	196 (65)
> 2		51 (26)	56 (53) ‡	107 (35)
Mean (sd)		2·3 (1·9)	3·0 (1·9) ‡	2·5 (1·9)
Wounded limb infection:				
Any Infection, n (%)		157 (79) ‡	46 (43)	203 (67)
Osteomyelitis		1	11	12
Duration of Hospital Stay, n (%)				
≤ 1 mo		87 (45)	52 (50)	139 (47)
> 1 - ≤ 3 mo		60 (31)	36 (35)	96 (32)
> 3 mo		46 (24)	15 (15)	61 (21)
Mean (sd), d		63 (66) †	48 (51)	58 (61·5)
Physiotherapy, n (%)				
Any		179 (90)	93 (88)	272 (89)
Duration				
	- ≤ 3 mo	85 (49) †	28 (31)	113 (43)
	- > 3 mo	88 (51)	63 (69) †	151 (57)

***Functional status and outcome***

Whole SuTra ^2^ population. Overall, 66% of patients were satisfied or very satisfied with the functional results at 1-year. The rate of satisfaction decreased between 1 and 2 years, in particular among amputees: at 2-year, it was 51% in the overall population (Table 4). Persistent pain was recorded in 62 % and 80 % of patients at 1-and 2-year respectively. Pain was significantly more frequent in patients with LP than in amputees at one year but not at 2-year. Mean pain intensity was greater at 2 years in patients with LP [Maximum pain intensity -VAS-, mean (sd): overall: 5·4 (2·2); A: 4·3 (2·1); LP: 6·0 (2·3); intergroup p<0·01]. About half the patients, but significantly more amputees, considered they were “cured” both at 1- and 2-year. Conversely, all the patients treated with a reconstructive approach would choose this management again, while 79% of amputees would prefer reconstructive treatment if amputation were not medically unavoidable. The majority of patients (85·5%) declared they were satisfied with the care they received. Two years after the earthquake, 30% of patients were working, significantly more LP than A; 23·5% were still living in a tent and 46% declared to have some difficulties to access to food; 25·5% would require additional surgical management, mainly stump revision or osteosynthesis material removal.

**Table 4: Patients’ outcome:** *Abbreviations: *LP: Patients with limb preservation; n: number; yr: year ^a ^: Population 1-year: Overall n=282, A n=188, LP n=94 ^b ^: Population 2-year: Overall n=235 , A n=152 , LP n=83^c^:Population 1-year (> 15 yr) : Overall n=243, A n= 157, LP n= 86 ; ^c1^ 19 missing values; ^c2^ among 199 not working, 10 missing values; ^c3^ 5 missing values; ^c4^ 7 missing values ^d^: Population 2-year (> 15 yr): Overall n=205, A n=141, LP n=75; ^d1^ 2 missing values; ^d2^ 1 missing value ‡: p<0·01; *: Comparison 2011 vs 2012 (intragroup); °: Comparison A vs LP (intergroup)

		Amputation	L P	Overall
Functional status: Satisfied or very satisfied, n (%)				
1-yr^a^		130 (69)	55 (58·5)	185 (66)
2-yr^b^		79 (52)*‡	41 (49)	120 (51)*‡
“Cured”, n (%)				
1-yr^a^		115 (61)	29 (31)°‡	144 (51)
2-yr^b^		101 (66)	23 (28)°‡	124 (53)
Resuming previous physical activities at 2-yr^b^		51 (34·5)	13 (16)	64 (27)
Persistent pain, n (%)				
1-yr^a^		106 (56)	70 (74·5)°‡	176 (62)
2-yr^b^		116 (76)*‡	71 (85·5)	187 (80)*‡
Satisfaction with overall care, n (%)				
2-yr^b^ ****		126 (83)	75 (90)	201 (85·5)
Need for referring at 2-yr^b^				
Overall n (%)^b^ ****		57 (37·5)	50 (60) ‡	107 (45·5)
Surgical		28 (18)	32 (38·5)	60 (25·5)
Rehabilitation		6 (4)	14 (17)	20 (8·5)
Prosthesis / orthosis		23 (15)	4 (4)	27 (11)
Need for psychological support at 2-yr^b^, n (%)		19 (12·5)	12 (14·5)	31 (13)
Patients’ theoretical preference for LP^c1^, n (%)		111 (79)	83 (100) ‡	194 (87)
Working status, n (%)				
1-yr:				
	- Work lost since the earthquake^c2^	76 (57)	26 (47)	102 (54)
	- Working^c3^	17 (11)	22 (26) °‡	39 (16)
2-yr: Working^d1^		33 (26) *‡	27 (37)	60 (30) *‡
Housing in a tent, n (%)				
1-yr^c3^		64 (42)	36 (42)	100 (42)
2-yr^d2^		34 (26) *‡	14 (19) *‡	48 (23·5) *‡
Not enough food, n (%)				
Prior the earthquake^c4^		14 (9)	7 (8)	21 (9)
1-yr^c4^		96 (64)	44 (52)	140 (59)
2-yr^d^		64 (49) *‡	31 (42)	95 (46) *‡
				

Amputees and prosthesis. At 1- and 2-year, 92 % and 96% of the LL amputees, 11% and 28% of the upper-limb (UL) amputees respectively had a prosthesis, which was used a mean of 9 hours and 11 hours a-day respectively. The first prosthesis was delivered within a mean of 136 days. The proportion of amputees satisfied with their prosthesis at 1- and 2-year was 66% and 75%, respectively. Disabling phantom limb pain was infrequent (18 out of 141: 13%).

Subpopulation of amputees and patients with limb preservation, matched for the main baseline characteristics. n those 46 patients (A: n = 23, LP: n=23) with lower limb injury, matched for age, sex, type of injury and number additional lesions, trends similar to those observed in the global population were noticed. The worsening of the perceived functional status between 2011 and 2012 was even more pronounced in amputees (satisfied/very satisfied: 1-yr: 87%; 2-yr: 22%) compared to patients with LP (satisfied/very satisfied: 1-yr: 65%; 2-yr 14: 61%) .

**Quality of life**

The variations in SF36 scores between 2011 and 2012 are shown in Figure 2, with a reference to a group of Swedish subjects with anterior cruciate ligament reconstruction^24^(Figure 3). At 1-year, the health-related quality of life was impacted in nearly all SF 36 domains (Figure 2). Between 2011 and 2012, meaningful positive changes were observed in all affected domains except for body pain, which was stable and for emotional role, which worsened, mostly in amputees. Mean (sd) physical and mental SF 36 subscales significantly increased from 57% (19) to 66·5% (11) and from 58% (20) to 62% (10) respectively in the overall population, with a similar magnitude across treatment groups for the physical subscale. The mental subscale improved in LP [(from 55% (20) to 62% (10)], but not in amputees [from 60% (20) to 62% (10)]. At 2-yr emotional and physical roles were more negatively impacted in this Haitian series than in the Swedish subjects with ACL reconstruction (Figure 3.), underlining the severity of both the initial wounds and their late consequences in the present cohort.

**SF 36 scores at 1-year and 2-year (dotted lines) in the overall population, A and LP:**
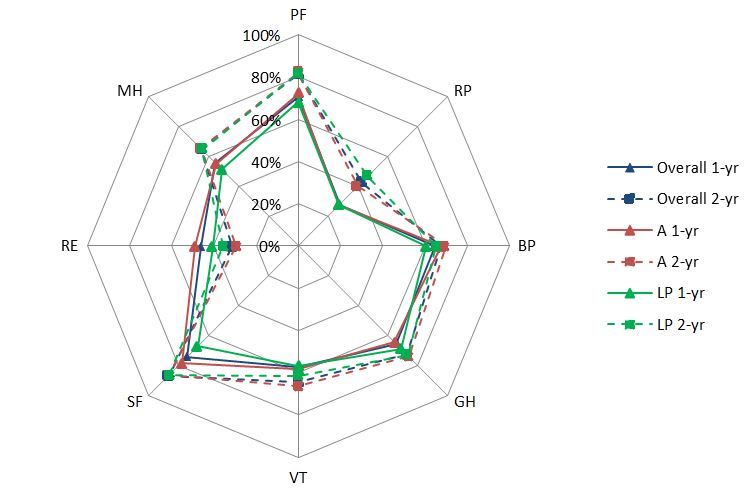
A: amputees; LP: Limb preservation; yr: year; mo: month; PF: Physical Functioning, RP: Role Physical, BP: Bodily Pain, GH: General Health, VT: Vitality, SF: Social Functioning, RE: Emotional Role, MH: Mental Health. Definitions:Physical subscale = mean of PF, RP, BP and GH; Mental subscale = mean of VT, SF, RE, MH; SF 36 score is improving from inner (0%) to outer (100%). At 1-year: overall n = 254, A: n= 161, LP: n= 93; At 2-year: overall: n = 204, A: n= 127, LP: n=77

>

**SF 36 scores at 1-year and 2-year in the overall population, with reference to Swedish subjects with anterior cruciate ligament (ACL) reconstruction at 6-month and 2-year*.**
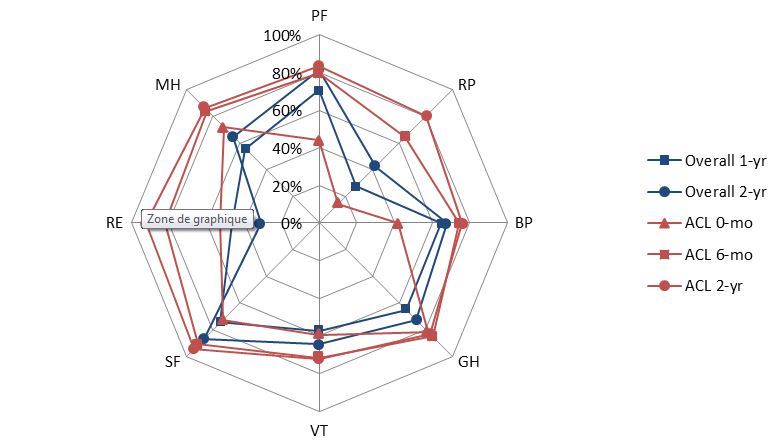
SF 36 score is improving from inner (0%) to outer (100%). * ACL: Swedish subjects,24: n = 62; mean age: 26 yr, male 80% (ref. 24)

## Discussion

Survivors of major trauma with orthopedic injuries especially lower limb injuries, usually have poor functional outcomes and quality of life^25^, particularly after a mass disaster in a developing country. The SuTra^2^study indicates that 2 years after the Haiti earthquake, only half the patients with limb injury, whether amputated or treated by conservative surgery, are satisfied with the functional results. The comparison of the outcome between 1 and 2 years shows a worsening of the perceived functional status while in parallel, the socio-economic status improved moderately. However at 2 years,only 30% of those victims with a job prior to the earthquake are working, 46% find access to food more or less problematic, and 23·5% are still living in a tent, a situation In Haiti known to be associated with negative outcomes for income, employment, and food access^16^.

As expected^10^ , ^26^ patients treated with conservative surgery were more frequently operated on than amputees. However, amputation was far from a straightforward procedure. The rate of stump revision was 30%, a figure in the range of those observed by others^4^ after the Haiti earthquake, far exceeding the 5.4% rate reported in the best, first world conditions^27^ . Furthermore, compared with patients with LP, amputees had a greater length of hospital stay. As observed in conventional medical settings^2^, amputation yields worse psychological outcomes, according to the SF36 scoring system, when compared to limb reconstruction. Compared to transtibial^28^ and transfemoral^29^ amputees retrospectively analyzed more than 28 years after the initial injury during the Vietnam War, quality of life impairments at 2-years in amputees after the Haiti earthquake were similarly with regards to the “role physical” (RP), but worse for the “role emotional” (RE) dimensions, indicating notable impairments due to physical limitations and their psychological consequences. However, contrary to Vietnam War amputees, the perceived health status of SuTra amputees (physical functioning – PF- ) was similar to controls. Although at 1 year amputees had better perceived functional outcomes than LP, and more amputees than patients with LP considered they were “cured” at 2 years, fewer amputees were at work at 2 years. Finally, most amputees (79%) would have preferred not to undergo amputation if it could have been avoided.

The SuTra^2^study population is representative of the population with severe limb injuries due to the Haiti earthquake reported by others^4^^,^ 7 . The study has included about 13% of all the amputees after the earthquake. The recruitment of amputees through organizations which mainly provide lower limb prostheses explains the lower rate of upper limb involvement (26%) in the SuTra^2^population, in comparison to the 36% rate reported by others^4^ , but a 37% ratio between UL and LL limb involvement was observed in the SuTra^2 ^patients with limb preservation. Amputations were performed earlier than conservative surgery (mean: day 6 post-earthquake). Inadequate numbers of specialized orthopaedic and plastic surgeons, not present yet in Haiti^4^ , and / or a lack of material resources, as well as the severity of the injuries may explain this early peak in amputation. Retrospective interviews among orthopedic surgeons who volunteered in Haiti within 30 days of the earthquake^19^ , suggested that inappropriate care may had occurred after the disaster. A considerable number of patients may have received primary amputation for complex injuries of limbs, which may have been salvageable^3^ . Indeed, the lowest rate of amputation has been reported in teams with a combination of orthopedic and plastic surgeons^4^ , ^10^. In the present series, a sizable number (29%) of victims undergoing conservative surgery had injuries which might have lead to amputation. In poor economies with minimal infrastructure and limited access to quality prostheses, the human and economic burden of limb loss can only be worse than in wealthy countries^10^.

The limitations of the study must be acknowledged. First, the lack of medical records and the heterogeneity of both the wounds and their initial treatment hampered analysis of outcomes in relation with the initial injuries and their management. This is a common drawback in reports on Haiti earthquake^4^ . Second, the mode of recruitment explains the higher proportions of amputees (65% of the patients), and of amputees younger than 15 years, compared to patients with LP, observed in the study. Finally, the 24% dropout rate between 2011 and 2012 should be seen in perspective, with poor living conditions and low socio-economic status for most patients. In the LEAP cohort conducted in a wealthy country, and followed for 7 years 26 ^,^ 30, the dropout rate at 2 years reached almost 20%. The wide distribution of mobile phones in Haiti, the word of mouth recruitment, and the reimbursement of the transport costs for attending the visits may have enhanced both the recruitment and the relatively high retention in the study in spite of the surrounding environment.

After a major earthquake, both the organization of emergency medical rescue to ensure optimal initial care^9^, and the long-term management of limb-injured victims are crucial for a favourable outcome. Despite inherent limitations, this study gives valuable information on the outcome of patients with severe limb injury after a mass catastrophe that can help prepare for future emergencies. First, notwithstanding a favourable outcome for amputees at one year, perceived functional status deteriorates with time, more rapidly than in patients with reconstructive management. Second, patients prefer limb preservation whenever possible. Third a sizeable proportion of amputees might have benefited from limb conserving treatment; in agreement with others^9^^,^ 10, wherever possible resources should be directed at limb salvage because of these potential long term benefits. Finally long term care and rehabilitation are mandatory for improving the outcome whatever the initial surgery performed, amputation or limb reconstruction, because the initial surgical procedure may have been sub-optimal, and the socio-economic context in developing countries is challenging. In mass disasters, postponing definitive surgery until adequate human and technical resources are available, or a transfer to tertiary referral centre is possible, may sometimes be the wisest decision^18^^,^ 31.Particular attention should be paid to clinical records, which should be handed to the patient^4^ . Guidelines for the overall management of limb injuries in mass casualties, as those established by Knowlton and colleagues^31^ for amputation, should be promoted. There is a professional and ethical obligation on those who provide humanitarian relief to achieve the best immediate outcomes possible in the circumstances, and also to recognize the long-term care, which will be needed to optimize outcomes for their patients.

## Author contributions

All authors participated in the literature search. Thierry Allafort-Duverger (TAD), Nikki Blackwell (NB), Stéphane Callens (SC), Marie C. Delauche (MCD), and Nezha Khallaf (NK) conceived the study concept and design. MCD in collaboration with SuTra^2 ^team members quoted in the acknowledgments acquired the data. Hervé Le Perff (HLP) in collaboration with SuTra^2^team members did the data processing. SC, NK, HLP, Joel Muller (JM), analyzed the data. All authors took part in their interpretation. NB, SC, MCD, HLP, NK, JM drafted the report, and all authors provided critical revisions and approved the final report. TAD NK, SC obtained funding from the he French Agence Nationale de Recherche.

## Competing interests

We declare no competing interests associated with any of the authors
